# Intraoperative breakage of disposable 27-gauge forceps resulting in retained intraocular metal fragment

**DOI:** 10.1016/j.ajoc.2022.101402

**Published:** 2022-02-09

**Authors:** Youning Zhang, Jay M. Stewart

**Affiliations:** a University of California, San Francisco, Department of Ophthamology, San Francisco, CA, USA; b Zuckerberg San Francisco General Hospital and Trauma Center, Department of Ophthalmology, San Francisco, CA, USA

**Keywords:** Surgical instruments, Intraocular foreign body

## 1 Case report

**Fig. 1:**
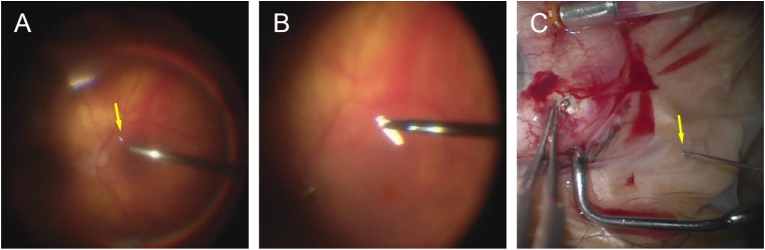
Retrieval of retained fragment. (A) Half of the forceps tip (arrow) was noted on the inferior retina. (B) 23-gauge forceps were used to retrieve the metal. (C) Removal of fragment (arrow).

A 76-year-old aphakic man underwent pars plana vitrectomy and scleral fixated lens placement. A 3-piece lens was inserted, and disposable 27-gauge MAXGrip forceps (Alcon, Fort Worth, TX) were used to externalize the haptics through 27-gauge cannulas 3 mm posterior to the limbus at 6 and 12 o'clock. During lens fixation, it was noted that half of the forceps tip was missing (Video 1). New forceps were used to complete the case; vision recovered to 20/30. One month later, the patient reported “one large floater.” A metallic object was noted inferiorly, along with a superotemporal retinal detachment with round holes at 11 and 12 o'clock without hemorrhages. The metallic object was identified as the missing forceps tip and was removed through an enlarged sclerotomy (Fig. 1; Video 2). The retinal detachment was repaired.

## 2Discussion

Our case represents the first description of intraoperative breakage of disposable 27-gauge instruments. Reviewing the original surgery, we recognized that when the 27-gauge cannula was pushed up the shaft of the forceps while being removed from its intrascleral location, the haptic was released from the grasp of the forceps, suggesting that it was during this maneuver that the tip broke. We presume that this occurred due to misalignment between the cannula and the forceps shaft/tip, as well as the extra stress put on the tip of the forceps which are only meant to peel fine membranes. Several cases of intraocular breakage of disposable retinal instruments have been described, all of which were 25-gauge.^1^^,^^2^ Inoue et al.^1^ speculated that breakage of a 25-gauge cutter tip was due to intrinsic fragility and mechanical manipulation. Our case helps raise an important message: certain surgical maneuvers are less forgiving with disposable small gauge instrumentation. The retained tip was not seen during evaluation of the retinal periphery, so it likely remained in the pars plana until it was subsequently released. As to the retinal detachment in our patient, although it is possible that the foreign body contributed to the detachment, we believe this was more likely related to vitreous traction and incarceration from the complicated forceps removal at 12 o'clock and reintroduction of a new forceps through the same sclerotomy, without a cannula, since the retinal holes were round, located in that same quadrant, and lacked any hemorrhages to suggest a traumatic impact.^3^

## 3Conclusion

Intraoperative breakage of disposable surgical instruments is uncommon and can be due to improper handling and intrinsic fragility.

## Intellectual property

XWe confirm that we have given due consideration to the protection of intellectual property associated with this work and that there are no impediments to publication, including the timing of publication, with respect to intellectual property. In so doing we confirm that we have followed the regulations of our institutions concerning intellectual property.

## Research ethics

XWe further confirm that any aspect of the work covered in this manuscript that has involved human patients has been conducted with the ethical approval of all relevant bodies and that such approvals are acknowledged within the manuscript.

IRB approval was obtained (required for studies and series of 3 or more cases)

Written consent to publish potentially identifying information, such as details or the case and photographs, was obtained from the patient(s) or their legal guardian(s).

Not applicable. Consent to publish the case report was not obtained. This report does not contain any personal information that could lead to the identification of the patient.

## Authorship

The International Committee of Medical Journal Editors (ICMJE) recommends that authorship be based on the following four criteria:

1. Substantial contributions to the conception or design of the work; or the acquisition, analysis, or interpretation of data for the work; AND
2. Drafting the work or revising it critically for important intellectual content; AND
3. Final approval of the version to be published; AND
4. Agreement to be accountable for all aspects of the work in ensuring that questions related to the accuracy or integrity of any part of the work are appropriately investigated and resolved.

## Contact with the editorial office

The Corresponding Author declared on the title page of the manuscript is:

## Patient consent

Written consent to publish this case has not been obtained. This report does not contain any personal identifying information.

## Funding

The authors would like to acknowledge financial support from 10.13039/100001818Research to Prevent Blindness and All May See Foundation.

## Authorship

All authors attest that they meet the current ICMJE criteria for Authorship.

## Declaration of competing interest

All authors have no disclosures to report.

## Appendix A Supplementary data

Video 1 Half of the forceps tip was missing as it was externalized through the superior sclerotomy. Video 1

Video 2 The superotemporal sclerotomy site was enlarged. 23-gauge forceps were used to remove the fragment. Video 2
